# Effects of Commitment on Fear of Failure and Burnout in Teen Spanish Handball Players

**DOI:** 10.3389/fpsyg.2021.640044

**Published:** 2021-03-18

**Authors:** Juan González-Hernández, Carlos Marques da Silva, Diogo Monteiro, Marianna Alesi, Manuel Gómez-López

**Affiliations:** ^1^Department of Personality, Evaluation and Psychological Treatment, University of Granada, Granada, Spain; ^2^Sport Sciences School of Rio Maior, Polytechnic Institute of Santarém Research Center in Life Quality (CIEQV), Rio Maior, Portugal; ^3^ESECS-Polytechnic of Leiria, Leiria, Portugal; ^4^Research Center in Sport, Health and Human Development (CIDESD), Vila Real, Portugal; ^5^Department of Psychological, Pedagogical and Education Sciences, University of Palermo, Palermo, Italy; ^6^Department of Physical Activity and Sport, Faculty of Sport Sciences, University of Murcia, Murcia, Spain; ^7^Campus of International Excellence “Mare Nostrum”, University of Murcia, Murcia, Spain

**Keywords:** sport commitment, self-criticism, shame, young athletes, emotional exhausted, depersonalization, reduced social realization, handball

## Abstract

Under an observational, transversal, and descriptive design, the study analyze the degree of adjustment of the perceptions of fear of failure as a mediating variable of the estimated relationship between sporting commitment and the appearance of burnout in young handball players in a competitive context. The sample included a total of 479 youth category handball players (250 boys and 229 girls) selected to compete in the Spanish Regional Championships. The age range was 16 (40.1%)−17 (59.9%) years old (*M* = 16.60; SD = 0.50). With regard to the years of experience variable, 85.4% stated that they have more than 5 years of experience at the federated handball player level. The Spanish version of *Performance Failure Appraisal Inventory* (PFAI), Inventory Athletes Burnout Revised (IBD-R), and Sport Commitment Questionnaire (SCQE) were used to assess fear of failure. The correlation patterns evidence that commitment is negative and significantly associated with emotional exhaustion, depersonalization, and fear of failure and positively associated with reduced sense of personal accomplishment. In the standardized direct effect, negative and significant effects were observed between commitment and fear of failure, fear of failure with emotional exhaustion and depersonalization, and on the contrary, a negative and significant effect was observed between fear of failure and reduced sense of personal accomplishment. The evaluated athletes reflect a positive psychological disposition, show pride in having been selected by their territorial teams and reflect a high desire to show their sporting qualities. Despite the emergence of cognitive-emotional processes associated with fear of failure (e.g., shame, fear of criticism), this has been observed to protect the sense of self-fulfillment through sport effort, although it also has impacts on further emotional exhaustion and loss of value of sport effort.

## Introduction

Fear of failure appears above all in a competitive sport environment, precisely because of the combination of the personal desire to achieve goals or tasks, and the cognitive uncertainty of being able to achieve them (Correia, 2018). Taking into account that most of the sport actions of young athletes are regularly evaluated under a performance and success criterion by external figures (Sagar et al., 2007), sport experience self-perception will be oriented toward the feeling of fear of making a mistake, when it is associated with the appearance of a feeling of shame (Gómez-López et al., 2019), ridicule (Eitzen, 2016), or embarrassment (Ellison and Partridge, 2012).

Due to this external evaluation and the excessive value that the player grants (especially during the game, and in the face of a decrease in performance), insecurity feelings, anxiety-stress, and avoidance behaviors will appear with more intensity (Moreno-Murcia and Conte, 2011). This fear of failure appears when the player focuses both attention and beliefs that others (e.g., peers, opponents) are responsible for controlling their behavior, seeking their approval, and/or fearing disapproval. According to Conroy et al. (2001) and Sagar et al. (2007), fear of failure emerges in childhood and increases with age. Therefore, failure itself would not have negative connotations if it were not for the cognitive over-evaluation (mainly of others) and aversive consequences that the athlete experiences toward his or her self-worth (Bélanger et al., 2013; Granz et al., 2019).

Surprisingly, fear has been associated with exhaustion on very few occasions in the scientific literature (Bicalho and Da Costa, 2018). Previous studies have highlighted that a high fear of failure is associated with higher levels of psychological suffering and a risk of burnout, supported mainly by avoidance of shame, embarrassment, or criticism (Lemyre et al., 2008). Fear of failure conceptualization as the tendency to evaluate the threat to the achievement of personal and significant goals contemplates that athletes have learned to associate failure with aversive consequences (Chen et al., 2008), making the aversive consequences grow after the anticipated failure (Correia, 2018), often coexisting with negative processes associated with anxiety and the gradual wearing out of their emotional response (Correia et al., 2016).

However, the multidimensional nature of both the psychological response to fear and sporting situations offers different interpretations as to the relationship of these variables (Gustafsson et al., 2016), taking into account the individual and not the contextual variables as a unit of analysis (Ruiz-Sánchez et al., 2017). From person-centered approaches (dynamic system of construction of the psychological response), it is understood that from personal stability, fear of failure (mainly fear of important others losing interest) is positively associated with greater emotional and physical suffering, although relationships are not as clear with indicators such as depersonalization (devaluation and withdrawal from sport) and reduced personal fulfillment (mainly, fear of experiencing shame and embarrassment; Gustafsson et al., 2017a). As we said, the interpretation as a constant negative reinforcement makes the fear of failure build a process of psychological hypersensitivity that emotionally wears out the athlete from his cognitive (e.g., rumination or worry) and emotional (e.g., anxiety, anger, guilt, or shame) areas (Correia, 2018). In a social situation such as competing in team sports, when members of a given group share perceptions of certain stressful or distressing events and contexts, competition is perceived as group agonistic and with poor use of psychological resources (e.g., low self-efficacy; Kozusznik et al., 2015; Gómez-López et al., 2020).

The scientific literature considers sport commitment as a motivating force that reflects the desire and determination to continue the sport effort (Scanlan et al., 1993; Weiss, 2020). Described as a dynamic psychological state that varies over time, seasons, or the course of competitions, it influences the persistence and function of sporting behavior for the athlete (Carpenter and Scanlan, 1998; Scanlan et al., 2003). As any psychological process of an internal nature, it also maintains links with its conceptual references, through the motivational orientation (mainly toward performance tasks) that both coaches and partners exercise on the athlete (Torregrosa et al., 2011). On the other hand, commitment in sportsmen and women has been considered a health protection factor in case of disruptive responses such as burnout or sport abandonment (Raedeke, 1997; Sousa et al., 2007), in relation to the valuation made on the costs or investment of effort (temporary and learning; Williams, 2013), so that the higher the commitment to sport practiced, the higher the value associated to the enjoyment and personal investment made, while a low commitment is associated to an overvaluation of costs and new alternative efforts.

Psychological commitment also involves a sustained effort to identify what sport represents. It means expressing behavioral consequences associated with the motivated behavior (e.g., being persistent, maintaining an intensity of effort, strengthening the intention to carry out tasks to achieve results) as a part of themselves (Zahariadis et al., 2006). In that way, athletes who feel more committed suffer more intensely from this hypersensitivity, as they magnify their goals by pursuing much higher standards, build unrealistic expectations, or feel too identified with what sport represents to them (Olusoga and Kenttä, 2017). From a functional-dysfunctional point of view, fear of failure configures a response differentially subjected to performance devaluation for the athlete, infusing concerns in the perceptions of lack of achievements and success (Madigan et al., 2016; González-Hernández and Muñoz-Villena, 2019), that is why the psychological commitment could be altered and deteriorated in the absence of a compensatory response (e.g., social support, positive coping) that balances the mentioned motivational process.

**Figure 1:**
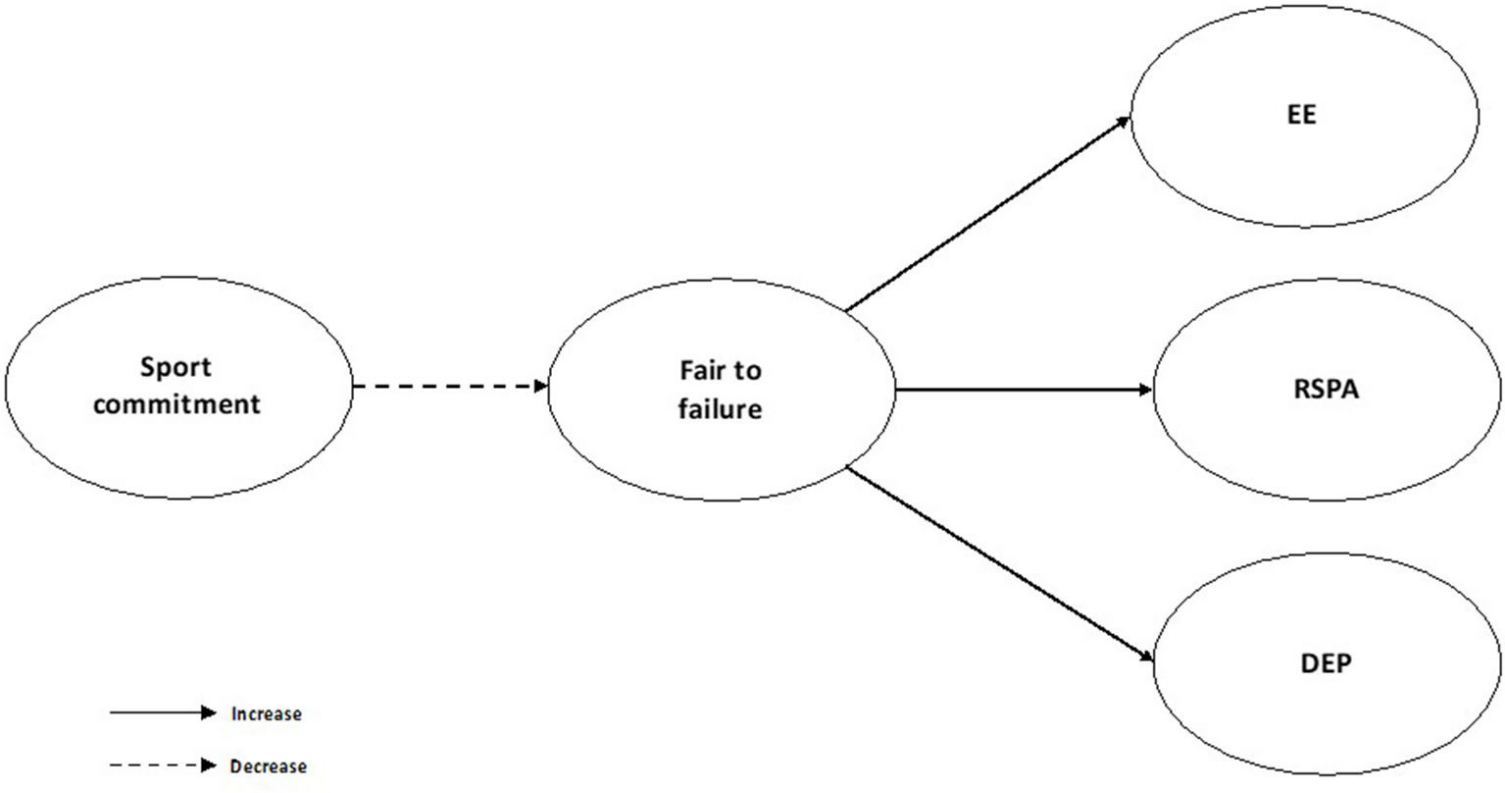
Standardized individual parameters—hypothesized model. EE, emotional exhaustion; RSPA, reduced sense of personal accomplishment; DEP, depersonalization.

With an observational and descriptive design of a transversal nature, the aim of this study was to analyze the degree of adjustment of the perceptions of the fear of failure as a mediating variable of the estimated relationship between sporting commitment and the appearance of burnout in young handball players in a competitive context, hoping to describe a hypothesized model (Figure 1) that reflected a negative effect of the devaluation of the fear of failure, capable of increasing the response of burnout in young athletes who show greater commitment. More specifically, the most committed athletes are also expected to show low indicators of fear of failure, emotional exhaustion, depersonalization, and low self-realization.

## Materials and Methods

### Participants

This study is part of the following project: Analysis of the factors implicit in the teaching-learning process of the handball player of the University of Murcia. Other Spanish universities such as Granada, Extremadura, and Almeria have collaborated in this project. It was developed with the consent of the Royal Spanish Handball Federation (RFEBM) and the Andalusian Handball Federation (FABM).

The sample included a total of 479 youth category handball players (250 boys and 229 girls) selected to compete in the Spanish Regional Championships (C.E.S.A.—Almería 2016). These selected players are the best handball players in their regions. They are the regional selections (17 men's and 16 women's teams), and most of them, due to their age, have participated in more than two Spanish handball championships. The same study population was used in both this study and in the following paper: Alesi et al. (2019). These players were rated “high-performance players” by the Spanish Sports Council according to Royal Decree 971/2007, of 13 July, on high-level and high-performance players. The age range was 16 (40.1%)−17 (59.9%) years old (*M* = 16.60; SD = 0.50). With regard to the years of experience variable, 85.4% stated that they have more than 5 years of experience at the federated handball player level.

### Measurement Instruments

Performance Failure Appraisal Inventory (PFAI) (Conroy et al., 2002) adapted to Spanish language by Moreno-Murcia and Conte (2011) was used. The scale includes 25 items grouped in five first-order dimensions [Fear of Experiencing Shame and Embarrassment (e.g., “When I am failing, it is embarrassing if others are there to see it.”), Fear of Devaluing One's Self-estimate (e.g., “When I am failing, it is often because I am not smart enough to perform successfully”), Fear of Having an Uncertain Future (e.g., “When I am failing, I believe that my plans for the future will change”), Fear of Important Others Losing Interest (e.g., “When I am not succeeding, some people are not interested in me anymore”), and Fear of Upsetting Important Others (e.g., “When I am failing, important others are disappointed”)] and one general dimension (fear to failure). All items were headed by the phrase “In my sports practice…” The answers were collected on a Likert-type scale ranging from do not believe at all (1) to believe 100% of the time (5). Here, the internal consistency analysis was satisfactory for the different subscales; Fear of Experiencing Shame and Embarrassment, α = 0.85; Fear of Devaluing One's Self-Esteem α = 0.70; Fear of Having an Uncertain Future, α = 0.83; Fear of Important Others Losing Interest, α = 0.84; Fear of Upsetting Important Others, α = 0.81.

Sport Commitment Questionnaire (SCQ; Scanlan et al., 1993), in its version adapted and validated to Spanish by Sousa et al. (2007) was used. This questionnaire consists of 32 items, maintaining a structure of seven dimensions of first order [Sport Commitment (e.g., “I will continue to play this sport for as long as I can”), Sport Enjoyment (e.g., “I love to play this sport”), Involvement Alternatives (e.g., “I would like to do other activities instead of practicing sport”), Personal Investments (e.g., “The time I have spent in this sport makes it difficult to stop playing”), Social Constraints (e.g., “People who are important to me expect me to keep playing this sport”), and Involvement Opportunities (e.g., “I would really miss the things I learn in this sport if I didn't play”)] and one general factor of second order. Each item is answered on a Likert-type scale ranging from 1 (strongly disagree) to 5 (strongly agree). In this study, we use a unique dimension commitment, showing a good internal reliability (α = 0.82).

Burnout Inventory for Athletes-Reduced (IBD-R; Garcés de Los Fayos et al., 2012). The IBD-R is a reduced version of Garcés de Los Fayos' 19-item IBD (1999), which comes from the Maslach Burnout Inventory (MBI; Maslach and Jackson, 1981). This Inventory evaluates burnout in athletes as a three-dimensional construct, characterized by Emotional Exhaustion (e.g., “Carrying out a work discipline in my sporting activity exhausts me”), Reduced Personal Realization (e.g., “I am effective in dealing with the problems of the people around me in the sporting environment”), and Depersonalization (e.g., “I don't really care what happens to the people around me in my sporting activity”). Each item is answered on a Likert-type scale ranging from 1 (I've never thought or felt it) to 5 (I think or feel it every day). To find the total score on each subscale, the scores of the items that make up that subscale are added together. The higher the score, the higher the level of burnout experienced by the athlete, except in the items of Reduced Personal Achievement which are formulated in the opposite direction: the lower the numerical response of the subject, the higher the degree of burnout experienced. In this study, the internal consistency values were: Emotional Exhaustion (α = 0.74), Reduced Personal Realization (α = 0.76), and Depersonalization (α = 0.72).

### Procedure

The study was carried out during the Spanish Championship of Autonomic Selections (CESA). The RFEBM, the FABM, and the coaches of the different regional selections all granted permission prior to our data collecting after reading a letter explaining the objectives of the study and the way it would be carried out. Prior to the administration of the questionnaires to the participants, and in accordance with the ethical guidelines of the American Psychological Association (APA), they were presented with an informed consent for ethical compliance and data protection, ensuring in this way, the rigor of the investigation and the privacy of the information obtained. The consent obtained from the players and their parents or tutors was both written and informed. A sample of the instrument was provided for them all. Data collection was carried out in Almeria during the Spanish Championship C.E.S.A. 2016, at the hotels where the teams were staying during players' time off, in agreement with the coaches and in the presence of one of the researchers. Each participant had 20–30 min to complete the questionnaire, and they were all briefed on the objects of the study and on their rights as participants, on the voluntary nature of the study, and on the confidentiality of answers and data management. They were also informed that there were no correct or incorrect answers and were asked to give true and honest replies. Following data verification, the following variables were recorded: gender, year of birth, years of experience as a handball player, playing position, and the numbers of hours per week dedicated to training, as well as the times it was carried out. This study was carried out in accordance with the ethical guidelines of the APA. Protocol was approved by the Ethics Committee of the Universidad de Murcia (ID: 1494/2017). All subjects gave written informed consent in accordance with the Declaration of Helsinki (World Medical Association, 2013).

### Analysis Data

Descriptive statistics, including means and standard deviation, as well as bivariate correlations were performed for all studied variables. A two-step maximum likelihood (ML) approach was performed in AMOS 23.0 as suggested by Kline (2016). Confirmatory factor analysis (CFA) was conducted to verify the psychometric properties of the measurement model (i.e., purposed model). During this stage, composite reliability, trough Raykov (1997) formula, was used to determine internal consistency, assuming 0.70 as a cut-off value, as suggested by Hair et al. (2014). In addition, convergent validity *via* average variance extracted, was performed, and values ≥0.50 were considered adjusted (Hair et al., 2014), while discriminant validity was established when the correlation coefficients were lower than the AVE for each construct that exceeded the squared correlations between that construct and any other (Fornell and Larcker, 1981).

A structural equation modeling (SEM) was made to test the purposed associations across different constructs. For both analyses, the traditional goodness-of-fit indexes were used: Comparative Fit Index (CFI), Normalized Fit Index (NLI), Standard Root Mean Residual (SRMR), and Root Mean Square Error of Approximation (RMSEA) with its Confidence Interval (CI: 90%). For these indices, scores of CFI and NLI ≥0.90 and SRMR and RMSEA ≤ 0.8 were considered acceptable, following several recommendations (e.g., Marsh et al., 2004; Byrne, 2010; Hair et al., 2014).

For mediation analysis, the direct and indirect effects among constructs on outcome variable were analyzed as suggested by Hair et al. (2014) and Hayes (2018). Bootstrap resampling procedure (1,000 samples) *via* AMOS 23.0 was performed through bias-corrected 95% CI to analyze the significance of direct and indirect effects. Indirect effect is considered significant (≤0.05) when its confidence interval does not include zero (e.g., MacKinnon et al., 2004; Williams and MacKinnon, 2008; Hayes, 2018). Effect sizes were classified as trivial (0–0.19), small (0.20–0.49), medium (0.50–0.79), and large (0.80 and greater), as suggested by Cohen (1992).

## Results

### Preliminary Analyses

No missing values or outliers (univariate or multivariate), as well as no univariate distribution violations were observed. Nevertheless, Mardia's coefficient form multivariate kurtosis has exceeded the recommended value (>0.5). Therefore, bootstrap Bollen-Stine (2,000 samples) was performed according to Nevitt and Hancock (2001). Additionally, variance inflation factor (VIF) was calculated to check the collinearity diagnosis. Results indicate that all VIF values were <10, a recommended value suggested by Hair et al. (2014). Finally, a GPower 3.1 (Faul et al., 2009) was used to determine the required sample size (considering the following parameters: effect size *f* ^2^ = 0.1; α = 0.05; statistical power = 0.95, and four predictors) pointing that the minimum required size would be 176 subjects, which was respected in the present study.

### Measurement Model

Means, standard deviations, and bivariate correlations among all constructs are presented in Table 1. In general, athletes exhibit high levels of commitment (*M* = 2.74; SD = 0.41), reduced sense of personal accomplishment (*M* = 2.82; SD = 0.45), moderate values of fear of failure (*M* = 2.08; SD = 0.79), and depersonalization (*M* = 2.29; SD = 0.78). In contrast, athletes showed lower levels of emotional exhaustion (*M* = 1.07; SD = 0.53).

**Table 1:** Descriptive and correlation analysis for all constructs and composite reliability.

**Constructs**	**COM**	**FF**	**EE**	**DEP**	**RSPA**
COM	–	–	–	–	–
FF	−0.33^**^	–	–	–	–
EE	−0.59^**^	0.76^**^	–	–	–
DEP	−0.29^**^	0.65^**^	0.79^**^	–	–
RSPA	0.46^**^	−0.27^**^	−0.27^**^	−0.21^**^	–
Mean	2.74	2.08	1.07	2.29	2.82
SD	0.41	0.79	0.53	0.78	0.45
CR	0.81	0.84	0.74	0.73	0.81

COM, commitment; FF, fear of failure; EE, emotional exhaustion; DEP, depersonalization; RSPA, reduced sense of personal accomplishment; CR, Composite reliability; SD, standard deviation;

** *p ≤ 0.01*.

The correlation patterns evidence that commitment was negative and significantly associated with emotional exhaustion, depersonalization, and fear of failure. In contrast, commitment was positive and significantly associated with reduced sense of personal accomplishment. On the other hand, fear of failure was positive and significantly associated with both depersonalization and emotional exhaustion and negative and significantly associated with reduced sense of personal accomplishment. Regarding burnout constructs, results showed that depersonalization and emotional exhaustion are associated negatively and significantly with reduced sense of personal accomplishment while reduced sense of personal accomplishment is positive and significantly associated with emotional exhaustion.

Finally, it was possible to observe that all constructs present an adjusted value of composite reliability, since all of them are ≥0.70 (Hair et al., 2014). The test of measurement model included commitment, fear of failure, and burnout dimensions (i.e., depersonalization, reduced sense of personal accomplishment, and emotional exhaustion). Results shown that measurement model fit to the data [χ^2^ = 412.91 (395); SRMR = 0.049; B-Sp = <0.001; RMSEA = 0.038 (90% CI = 0.032, 0.045); TLI = 0.926; CFI = 0.935]. Additionally, measurement model revealed no problems in both convergent and discriminant validity, since the average variance extracted was ≥0.50 (Fornell and Larcker, 1981; Hair et al., 2014) and the square correlations among all constructs are less than the AVE of each factor (Fornell and Larcker, 1981).

### Structural Model

**Figure 2:**
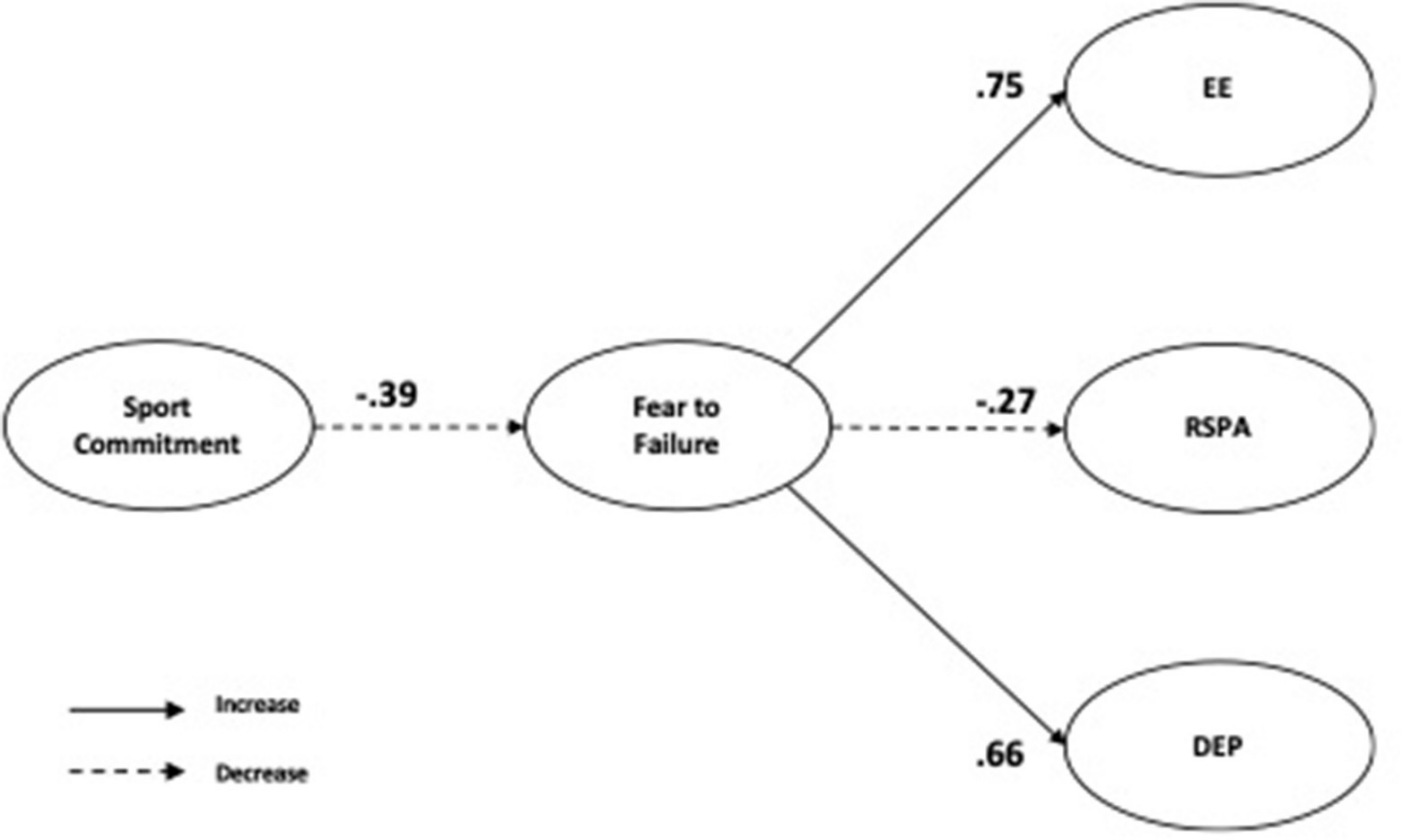
Standardized individual parameters—Hypothesised model. Note: EE, emotional exhaustion; RSPA, reduced sense of personal accomplishment; DEP, depersonalization.

The structural model shown a good fit to the data [χ^2^ = 499.04 (401); SRMR = 0.061; B-Sp = <0.001; RMSEA = 0.046 (90% CI = 0.040, 0.052); TLI = 0.900; CFI = 0.910]. In the standardized direct effect (Figure 2), negative and significant effects were observed between commitment and fear of failure (*β* = −0.39 [−0.485, −0.279]). In addition, fear of failure showed a positive and significant effect with both emotional exhaustion (*β* = 0.75 [0.663, 0.821]) and depersonalization (*β* = 0.66 [0.568, 0.738]). In contrast, a negative and significant effect was observed between fear of failure and reduced sense of personal accomplishment (*β* = −0.27 [−0.382, −0.143]) (Figure 2).

Regarding mediation analysis between commitment, through fear of failure, on depersonalization, emotional exhaustion, and reduced sense of personal accomplishment, results show a negative and significant indirect effect between commitment in both depersonalization (*β* = −0.25 [−0.335, −0.173]) and emotional exhaustion (*β* = −0.29 [−0.382, −0.196]), and a positive and significant effect between commitment reduced sense of personal accomplishment (*β* = 0.10 [0.052, 0.168]) *via* fear of failure. In sum, the mediation analysis revealed that fear of failure mediates negatively the association between commitment in both depersonalization and emotional exhaustion. In contrast, fear of failure mediates positively the association between commitment and reduced sense of personal accomplishment.

## Discussion

The aim of the study was to analyze the degree of adjustment of the perceptions of the fear of failure as a mediating variable in the estimated relationship between sporting commitment and the appearance of burnout in young handball players in a competitive context.

In the first instance, we sought to show the linear relationship between the variables under study, describing in the line of previous works that the protective value of sport commitment on the fear of failure (Bélanger et al., 2013) and burnout (Raedeke, 1997; Sousa et al., 2007; Williams, 2013). However, in a less expected way, as recent similar studies have pointed out (Woods et al., 2020), the commitment was positive and significantly associated with a lesser sense of self-fulfillment. As expected, fear of failure was positively related to burnout indicators (Lemyre et al., 2008; Correia et al., 2016; Gustafsson et al., 2016).

In the hope of reflecting the negative effect of cognitive devaluation caused by fear of failure on sporting commitment, and its corresponding orientation toward the occurrence of burnout, a model was tested which proposed the following sequence: sporting commitment, fear of failure, and consequences of burnout. As suggested in the literature reviewed (Chen et al., 2008; Bélanger et al., 2013; Correia et al., 2016), a clear reverse effect of commitment vs. burnout was shown through the emergence of fear of failure (Gustafsson et al., 2016). As expected, emotional and cognitive depreciation aimed at the negative consequences that accompany making mistakes increases the occurrence of both emotional exhaustion and depersonalization (Williams, 2013; Kozusznik et al., 2015). However, it has only been possible to partially fulfill the hypotheses put forward, as negative links have been observed between fear of failure and a reduced sense of accomplishment.

Different studies reflect that those athletes with a high level of sport commitment enjoy higher levels of motivation (Ryan and Deci, 2000; Pulido et al., 2018), increasing their enjoyment (Schmidt and Stein, 1991; Tamminen et al., 2016) and involvement (Funk et al., 2011) with sports practice. In addition, sportsmen and women with a high level of sporting commitment increase team performance (Gross et al., 2018), collective identity (Wann and Pierce, 2003), or the perception of task- and care-oriented climates among team members (Torregrosa et al., 2011; Hall et al., 2017). On the contrary, and in the same way as the results of the present work, engagement already in young athletes decreases the anxious perception of competitive situations (Pons et al., 2018) and enhances positive emotions (McCarthy, 2011; Friesen et al., 2013) in young athletes.

With regard to its links with burnout, sport commitment has maintained negative relationships with both emotional exhaustion, sports devaluation, and reduced personal fulfillment throughout most of the scientific literature (Gustafsson et al., 2017b; Bicalho and Da Costa, 2018) both in individual (Weiss et al., 2001) and team sports (Cresswell and Eklund, 2006; Curran et al., 2013) and in professional sports (Hill et al., 2008) or in lower categories (Harris and Watson, 2011; Pons et al., 2018). However, in team sports, it has been argued in recent studies that being committed to the team is linked to an increased deterioration in sporting self-fulfillment, essentially when the pride and honor associated with belonging to a team is present, often combined with a sense of responsibility and a desire to maintain standards of performance in order to contribute and be useful to the team (Woods et al., 2020).

Precisely, many of these elements associated with commitment, also linked to the paths that lead to focus on the fulfillment of objectives (e.g., wanting to show the group usefulness, playing the assigned role; Weiss, 2020), seem to have strong positive effects that cannot be limited by the appearance of fear of failure (Hughes and Hassan, 2015).

Research indicates how the enthusiasm and challenge with which one hopes to overcome a challenge, the pride in representing one's team or sport (e.g., national team), or the sacrifice required to identify with success (Lanter and Hawkins, 2013; Woods et al., 2020).

Apart from the fact that the study carried out offers an important contribution to the way in which the commitment generated in young sportsmen and women by a team sport such as handball can be linked, it is necessary to reflect some limitations with which to take into account the results obtained, mainly so that they can be taken into account in future similar proposals. In the correlational study, relationships are established between the variables, and there is no causal relationship. Although, correlation analyses provide an explanatory model that allows a better and more comprehensive understanding of the relationship between sport commitment, fear of making mistakes, and burnout indicators. The resulting model, taking into account the problem of equivalent models presented by the structural equation technique (Hershberger and Marcoulides, 2006), is assumed to be one more of the possible models in this study. Although the sample size is considerable, it would be more conclusive with a larger sample that could also provide studies on invariance on some sociodemographic conditions (e.g., gender, age, etc.).

Aware of the limitations of descriptive studies, we assume the need to carry out more studies in this line that contrast the results obtained with other groups of athletes (e.g., individuals vs. team; different sports transitions) or from a cross-cultural perspective. In this way, it would be necessary to complement future studies with the analysis of the influence they exert (e.g., coaches, peers) on the relevance of sport commitment and the protective or risk value of fear of failure for burnout indicators. Another interesting aspect would be to contrast the perception that athletes have under the influence of different variables in which it has been evidenced that they maintain a link on their representation as athletes (e.g., perfectionism, impulsivity). Also, to explore the links that fear of failure, sporting commitment, and burnout maintain with internal variables of the condition of athletes (e.g., self-esteem, self-concept) or emotional response (e.g., anxiety, emotional intelligence) in terms of their experiences of success and failure. Finally, the proposal of new models that complement and complete the results obtained, will allow to configure an extended scientific background that offers more information for the resources applied in psychological training.

## Conclusions

Regarding the mediation analysis between commitment, fear of failure, depersonalization, emotional exhaustion, and reduced sense of personal accomplishment, results show a negative and significant indirect effect between commitment in both depersonalization and emotional exhaustion and a positive and significant effect between commitment-reduced senses of personal accomplishment, *via* fear of failure. In sum, the mediation analysis revealed that fear of failure mediates negatively the association between commitment in both depersonalization and emotional exhaustion. In contrast, fear of failure mediates positively the association between commitment and reduced sense of personal accomplishment.

The evaluated athletes reflect a positive psychological disposition, which is synonym with desire and decision to contribute to their teams, participating and adding to the competition (they are young people selected for their sporting talent to represent their territorial teams). Despite the appearance of cognitive-emotional processes associated with fear of failure (e.g., shame, fear of criticism), it has been observed that this constitutes a way of protecting the sense of personal fulfillment through sporting effort. However, this same component, centered on the devaluation of the capacities for sporting action and the overestimation of the negative consequences of the mistake, becomes a risk factor for emotional exhaustion and the distancing/withdrawal of the sporting value.

From the perspective of sport commitment models, which are closely associated with achievement motivation, it is possible to link elements that facilitate positive adaptation (for example, the degree of enjoyment, the valuation of personal investments, or participation opportunities), committing personal efforts to a markedly successful path in the competitive environment. Young athletes who aspire to be elite, more susceptible, and ambitious to reach higher levels or to demonstrate their talent, place a high value on the styles of their coaches, on the links with their peers, and on the details that lead them to improve their sporting opportunities, while overstating those aspects that can lead them to make mistakes, dangerously linked to perceptions of failure (almost equally perceived).

It is of great relevance that professionals of sport training and sport psychology design possibilities to experience behaviors of fear of failure linked or inserted in positive coping strategies toward sport action. In this way, the adaptive process will focus on improving the perception of competence and personal (e.g., self-confidence) and psychosocial (e.g., leaning on others) resources to be put into action at every moment of sport performance. The prelude to success is the functional learning of “not afraid to fail.” Otherwise, as performance-oriented avoidance behavior that focuses on all the negative aspects of failure (e.g., external criticism, cognitive and emotional devaluation, medium- and long-term underperformance), it will make the sports experience an agony rather than an escape.

## Data Availability Statement

The raw data supporting the conclusions of this article will be made available by the authors if they are requested, and without undue reservation (jgonzalez@ugr.es).

## Ethics Statement

The studies involving human participants were reviewed and approved by the Ethics Committee of the Universidad de Murcia (ID: 1494/2017). Participants and participants' legal guardian/next of kin provided their written informed consent to participate in the study.

## Author Contributions

JG-H and MG-L: conceptualization, data curation, and writing—review and editing. DM and CdS: methodology and formal analysis. MG-L: investigation. All authors writing-original draft preparation and supervision.

## Conflict of Interest

The authors declare that the research was conducted in the absence of any commercial or financial relationships that could be construed as a potential conflict of interest.
